# Grazing Changed Plant Community Composition and Reduced Stochasticity of Soil Microbial Community Assembly of Alpine Grasslands on the Qinghai-Tibetan Plateau

**DOI:** 10.3389/fpls.2022.864085

**Published:** 2022-05-23

**Authors:** Yu Li, Shikui Dong, Qingzhu Gao, Chun Fan, Moses Fayiah, Hasbagan Ganjurjav, Guozheng Hu, Xuexia Wang, Yulong Yan, Xiaoxia Gao, Shuai Li

**Affiliations:** ^1^School of Public Administration, Chongqing Technology and Business University, Chongqing, China; ^2^School of Grassland Science, Beijing Forestry University, Beijing, China; ^3^Department of Natural Resources, Cornell University, Ithaca, NY, United States; ^4^Institute of Environment and Sustainable Development in Agriculture, Chinese Academy of Agricultural Sciences, Beijing, China; ^5^Department of Forestry, School of Natural Resources Management, Njala University, Njala, Sierra Leone; ^6^Beijing Academy of Agricultural and Forestry Sciences, Beijing, China; ^7^China New Era Group Corporation, Beijing, China; ^8^State Key Laboratory of Water Environment Simulation, School of Environment, Beijing Normal University, Beijing, China

**Keywords:** grazing regime, dominant species, functional composition, co-occurrence network, community assembly

## Abstract

Grazing is a substantial threat to the sustainability of grassland ecosystems, while it is uncertain about the variety of plant and soil microbial community and the linkages between them limit the comprehensive understanding of grazing ecology. We conducted an experiment on the effects of the grazing regimes rotational grazing (RG), continuous grazing (CG), and grazing exclusion (GE) on an alpine meadow in Qinghai-Tibetan Plateau. The differences of plant community composition, soil microbial community assembly mechanism, and taxonomic and functional composition between grazing regimes were examined, and the relationship between plant species and the soil microbes was assessed by constructing a co-occurrence network. The results showed that the plant community composition varied with the grazing regimes, while the soil microbial community composition did not vary with the grazing regimes. The soil bacterial functional composition was similar under RG and CG, while the soil fungal functional composition was similar under GE and RG. The soil microbial community under all grazing regimes was assembled mainly according to stochastic rather than deterministic mechanisms, and RG and CG reduced the relative importance of the stochastic ratio. At the microbial phylum level, CG and GE increased the relative abundance of Acidobacteria and Armatimonadetes and CG and RG increased the relative abundance of Elusimicrobia. In the network of plant species and soil microbial classes, plants and bacteria themselves were mainly positively linked (symbiosis and promotion), while plants and soil microbes were mainly negatively linked (competition). There were five microbial generalists in the network, which connected with many microbes, and four showed no difference in their abundance among the grazing regimes. Overall, the stable key microbes in the network and the fact that many of the plants are unconnected with microbes weakened the impact of grazing-induced changes in the plant community on soil microbes, probably resulting in the stable soil microbial community composition. Moreover, there was still a dominant and tolerant plant species, *Kobresia pygmaea*, that connected the plant and microbial communities, implying that the dominant plant species not only played a crucial role in the plant community but also acted as a bridge between the plants and soil microbes; thus, its tolerance and dominance might stabilize the soil microbial community.

## Introduction

Grassland ecosystems, accounting for approximately 40% of the terrestrial surface in China, are facing remarkable sustainability challenges from livestock grazing (Zhou et al., 2017; Tang et al., 2019). Therefore, an in-depth understanding of the impact of grazing on the grassland ecosystem is urgently needed to deal with and recover the grazing-induced grassland degradation. Grazing has substantial influences on grassland ecosystems such as the deposition of herbivore urine and dung, general decreases in soil porosity through trampling, changes in litter quality, and stimulated root exudates via selective defoliation (Zhong et al., 2018; Tang et al., 2019); these factors affect the nutrient sources and living environment of soil microbes. Conversely, the soil microbial community mediates the energy and material fluxes of ecosystems and alters soil biogeochemical properties (Chapin III et al., 2000), thus affecting the soil nutrients needed by the plant community. These linkages form a feedback response between the plant and soil microbial communities to grazing. In addition, a given plant species may preferentially associate with distinct soil microbial taxa (Fierer, 2017). As a result, understanding the effects of grazing on grasslands should stress not only the changes of the plant community or soil microbial community separately but also the linkages between them.

At present, the respective effects of grazing on plant community and soil microbial community are well understood (Shen et al., 2013; Yang et al., 2013; Beck et al., 2015; Macdonald et al., 2015; Wang et al., 2016; Xun et al., 2018; Tang et al., 2019). However, the relationship between plant and soil microbial community in grazed grasslands remains unclear, hence it hinders the systematic understanding of the effect of grazing on grassland ecosystems. To fill this knowledge gap, we conducted a grazing experiment on the grassland ecosystem of the Qinghai-Tibetan Plateau (QTP), the highest plateau in the world with a total area of 2.5 × 10^6^ km^2^ and an average altitude of over 4,000 m. As one of the key types of grassland on the QTP, the alpine meadow contributes considerably to global soil C and N pools (Li et al., 2014; Ding et al., 2016), and this grassland is also a very sensitive eco-region for climate change and anthropogenic disturbances (Tang et al., 2019) and is highly threatened by grazing (Li et al., 2020). The key objectives of the study were to explore (1) the effect of grazing on plant community composition; (2) the effect of grazing on soil microbial diversity, taxonomic and functional composition, and community assembly; (3) the relationship between plant community and soil microbial community in grazed grassland.

## Materials and Methods

### Study Area and Experimental Design

The grazing experiment was conducted in Nagqu city, Tibetan Autonomous Region of China, which is situated at the central QTP with latitude 31.441°N, longitude 92.017°E, and altitude 4,500 m a.s.l. The region has an arid and cold climate. The annual average temperature is 0.03°C and the annual average rainfall is 474.7 mm. In the growing season (May–September), the average rainfall is 414.7 mm and the average temperature is 7.8°C. The soil agro-type is alpine meadow soil with high proportions of clay. The main vegetation is an alpine meadow, with the plant community dominated by *Kobresia pygmaea* and accompanied by *Kobresia humilis, Potentilla humilis, Potentilla saundersiana, Potentilla bifurca, Astragalus membranaceus, Leontopodium leontopodioides, Stipa capillata*, and so on.

During the growing season of 2014–2017, the grazing experiment was carried out in a 30,000 m^2^ paddock of the alpine meadow, which was enclosed with wire mesh and divided into 12 50 × 50 m plots (Supplementary Figure S1). Nine plots were randomly placed on the paddock for rotational grazing (RG) and were further divided into groups A, B, and C, with each group having three replicates. The other three plots which were subjected to grazing before 2014 were used as three replicates of grazing exclusion (GE). At the beginning of July 2014, 2015, 2016, and 2017, two yaks with an average weight of 105±10 kg were grazed in each replicate plot of group A for 7 days (six yaks in three replicates). Then, these yaks grazed in groups B and C in turn in the same way. Three rotation cycles lasted 63 days each year except for 2015, in which only two rotation cycles lasted 42 days due to drought, which led to the earlier withering of plants. Three plots of the same size as the RG and GE plots were randomly selected as continuous grazing (CG) plots outside the fence in 2016 and 2017. The area outside the fence contained a 37.5-ha paddock of the alpine meadow that was permanently grazed by approximately 300 yaks. In the RG and CG plots, the grazing intensity was eight young yaks per hectare.

### Measurement of Soil and Plant Properties

On September 5, 2017, plant leaves of all species were collected following the method of Cornelissen et al. (2003) and Pérez-Harguindeguy et al. (2013). The sampling method has been specifically described previously (Li et al., 2019, 2020). In brief, at least 30 plants of each species were selected in each grazing regime; three to five fully expanded leaves of each individual were sampled for further leaf trait measurements. In the laboratory, the dry plant leaves of each species were ground by a ball mill (NM200; Retsch, Haan, Germany) for chemical analysis. Total carbon and nitrogen contents were measured by an elemental analyzer (Perkin-Elmer, Boston; MA, USA), and total phosphorus content was measured by an ICP-AES analyser (Thermo-Jarrell Ash Corp; MA, USA). In addition, six soil samples with a diameter of 5 cm were randomly collected at a depth of 0 to 15 cm in each plot, three samples were used to analyze the soil nutrient content and microbial community, and three samples were used to estimate the plant belowground biomass. The NH_4_-N and NO_3_-N of the soil samples were determined by a flow injection AutoAnalyser (AACE, Germany), and available phosphorus was determined by inductively coupled plasma spectrometry (SPECTRO ARCOS EOP, Germany). Soil total carbon content was measured using an elemental analyser (2400 II CHNS/O Elemental Analyser; Perkin-Elmer, Boston, MA, USA). Soil bulk density was determined with a cutting ring by sampling three cores in each plot at a depth of 0 to 15 cm. The detailed estimation of the aboveground and belowground biomass of the plant community can be found in our previous study (Li et al., 2020).

### Soil Microbial Community Analysis

The soil microbial community was analyzed using high-throughput sequencing. Soil microbial DNA was extracted from each soil sample using a Fast DNA SPIN Kit for Soil (MP Biochemicals, Solon, OH, USA). The V4-V5 of bacterial 16S rRNA gene was amplified by polymerase chain reaction (95°C for 2 min, followed by 25 cycles at 95°C for 30 s, annealing at 55°C for 30 s, and extension at 72°C for 45 s) using the universal primer 515F (5′-GTGCCAGCMGCCGCGG-3′) and 907R (5′-CCGTCAATTCMTTTRAGTTT-3′). ITS rRNA of fungi was amplified by polymerase chain reaction (95°C for 2 min, followed by 32 cycles at 95°C for 30 s, annealing at 61°C for 30 s, and extension at 72°C for 45 s) with primer ITS1F (5′-CTTGGTCATTTAGAGGAAGTAA-3′) and ITS2R (5′-GCTGCGTTCTTCATCGATGC-3′). The PCR products were gel-purified with an AxyPre DNA Gel Extraction kit (Axygen Biosciences, Union City, CA, USA) and quantified by Qubit® 2.0 Fluorometer (Invitrogen Corp., Carlsbad, CA, USA), then a mixture of amplicons was used for sequencing on the Illumina MiSeq platform. The processes of controlling quality and trimming sequencing reads were performed following the method of Hong et al. (2015). Operational Taxonomic Units (OTUs) were built at 97% sequence similarity cutoff using UPARSE (Edgar et al., 2011). OTUs were assigned to taxonomic lineages using the Ribosomal Database Project (RDP) classifier within the Silva database (http://www.arb-silva.de). The total sequence number in each sample was rarefied to the minimum sequence number across all the samples. The rarefaction curves and the number of OTU reads of samples are shown in Supplementary Figure S6 and Table S3, respectively. The functional profiles of soil bacteria and fungi were assigned by FAPROTAX 1.2.3 (Louca et al., 2016) and FUNGuild (Nguyen et al., 2016), respectively.

### Statistical Analyses

Two-tailed paired *t*-tests and analysis of variance (ANOVA) were performed to test the grazing regime effect on plant species coverage and soil total carbon content. Permutational multivariate analysis of variance (PERMANOVA) based on Bray–Curtis dissimilarity indices were performed to examine the difference in plant community composition among grazing regimes. Kruskal–Wallis tests were performed to examine grazing regime effects on soil microbial diversity indices and microbial phyla relative abundance. Microbial community alpha diversity was represented by richness, Shannon, Simpson, PD whole tree, and Chao 1 indices. Richness was presented as the observed OTU number. Three nonparametric tests (multiple response permutation procedure, MRPP; PERMANOVA; and analysis of similarity, ANOSIM) based on Bray–Curtis dissimilarity indices were performed by R software to examine the difference in soil microbial taxonomic composition among grazing regimes. A phylogenetic tree was annotated and visualized in iTOL software (Letunic and Bork, 2019).

A null modeling-based approach by Ning et al. (2019) was performed to infer soil microbial community assembly mechanism, that is, calculating the normalized stochastic ratio for estimating the relative importance of stochasticity and determinism in shaping community structure. Specifically, the null model algorithm was used to qualify the significance of the observed difference in the microbial community from random expectation. If the observed microbial community is statistically different from the null expectation, the community is regarded as largely shaped by deterministic processes. Otherwise, it is considered to be dominated by stochastic processes. Also known as normalized stochasticity ratio with 50% as the boundary point between more deterministic (<50%) and more stochastic (>50%) assembly.

To explore the relationships among bacteria, fungi, archaea, and plant species, a co-occurrence network based on the relative abundance of microbial classes and plant species dominance data was constructed using the MENA pipeline (http://ieg2.ou.edu/MENA), which performs random matrix theory to identify thresholds for constructing a highly confident ecological network (Zhou et al., 2011; Deng et al., 2012), and the default settings of MENA were adopted.

## Results

### Changes of Plant Community Composition With Grazing Regimes

**Figure 1:**
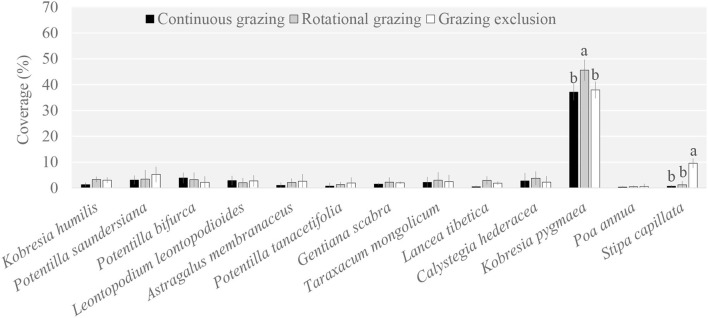
Plant species coverage among grazing regimes. Different lowercase letters indicate significant differences between grazing regimes (*p* < 0.05).

The plant community composition under RG was significantly different compared to that under GE and CG (Supplementary Table S1). Specifically, *Kobresia pygmaea* was the dominant species among the 13 main species; it was a palatable species for yaks and its coverage under RG was higher than that under GE and CG (Figure 1). Another priming species was *Stipa capillata*, which was also a palatable species. However, its coverage under GE was higher than that under RG and CG.

### Changes in Soil Microbial Diversity, Taxonomic, and Functional Composition With Grazing Regimes

**Figure 2:**
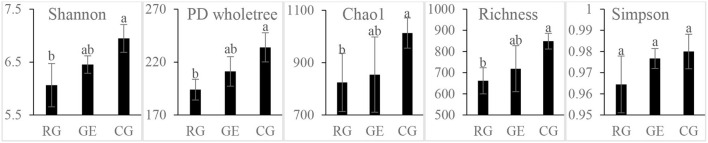
Soil fungal diversity among the grazing regimes. GE, grazing exclusion; RG, rotational grazing; and CG, continuous grazing. Different lowercase letters indicate significant differences between grazing regimes (*p* < 0.05).

**Figure 3:**
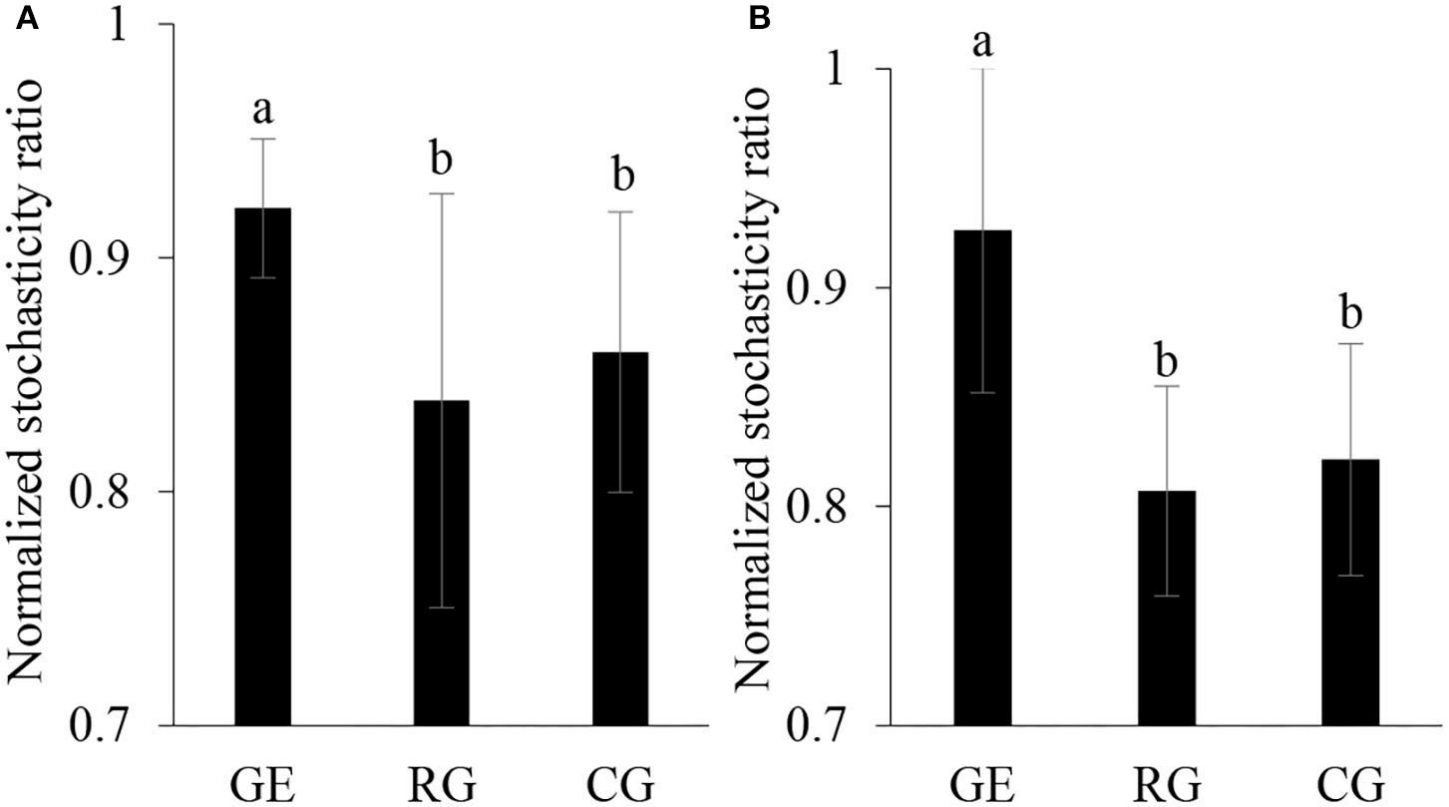
Normalized stochasticity ratio of community assembly of soil bacteria **(A)** and fungi **(B)**. GE, grazing exclusion; RG, rotational grazing; and CG, continuous grazing. Different lowercase letters indicate significant differences between grazing regimes (*p* < 0.05).

The soil bacterial diversity indices showed no differences among the grazing regimes (Supplementary Figure S2). The Shannon, Chao 1, richness, and PD whole tree indices of the soil fungal community were all higher under CG than under RG, while the Simpson index showed no difference among the grazing regimes (Figure 2). The soil's whole microbial community composition did not show any difference among the grazing regimes (Table 1). Stochasticity dominated soil microbial community assembly, RG, and CG significantly decreased the relative importance of stochastic processes (Figure 3).

**Table 1:** Nonparametric analyses to test the dissimilarity of the soil microbial communities among the grazing regimes.

		**ADONIS**		**ANOSIM**		**MRPP**	
		***F***	***p***	***R***	***p***	**δ**	***P***
Bacteria	RG-GE	1.630	0.087	0.258	0.113	0.323	0.146
	RG-CG	1.276	0.162	−0.123	0.707	0.286	0.038
	GE-CG	1.612	0.100	0.370	0.100	0.293	0.100
Fungi	RG-GE	0.902	0.645	−0.138	0.751	0.750	0.576
	RG-CG	1.094	0.289	**–**0.305	0.954	0.716	0.108
	GE-CG	1.249	0.200	0.222	0.200	0.676	0.200

*Three different permutation tests were performed, including the multiple response permutation procedure (MRPP), analysis of similarity (ANOSIM), and permutational multivariate analysis of variance (PERMANOVA), calculated with Bray–Curtis dissimilarity. GE, grazing exclusion; RG, rotational grazing; and CG, continuous grazing*.

**Figure 4:**
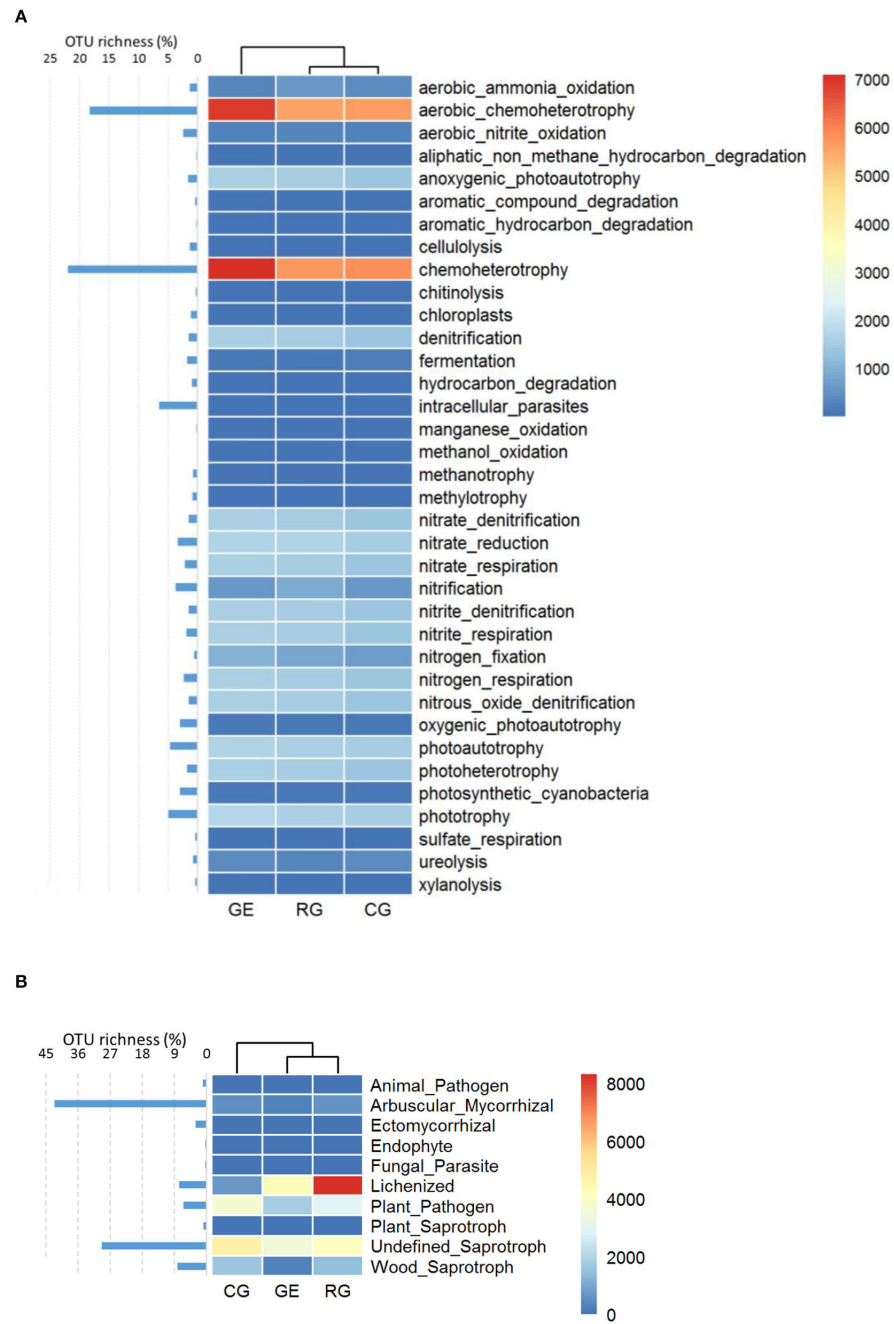
The relative abundance and OTU richness of bacterial **(A)** and fungal functional guilds **(B)**. GE, grazing exclusion; RG, rotational grazing; and CG, continuous grazing.

The soil bacterial functional composition under RG and CG was similar (Figure 4A), while the soil fungal functional composition under GE and RG was similar (Figure 4B). Specifically, aerobic chemoheterotrophy and chemoheterotrophy were the main bacterial functional guilds, which include most OTU, and their relative abundances were higher under GE than those under RG and CG. Lichenized and Undefined Saprotroph were the main fungal functional guilds, while most OTU belonged to Arbuscular Mycorrhizal, with low relative abundance.

### Changes in Dominant Soil Microbial Taxa Abundance With Grazing Regimes

**Figure 5:**
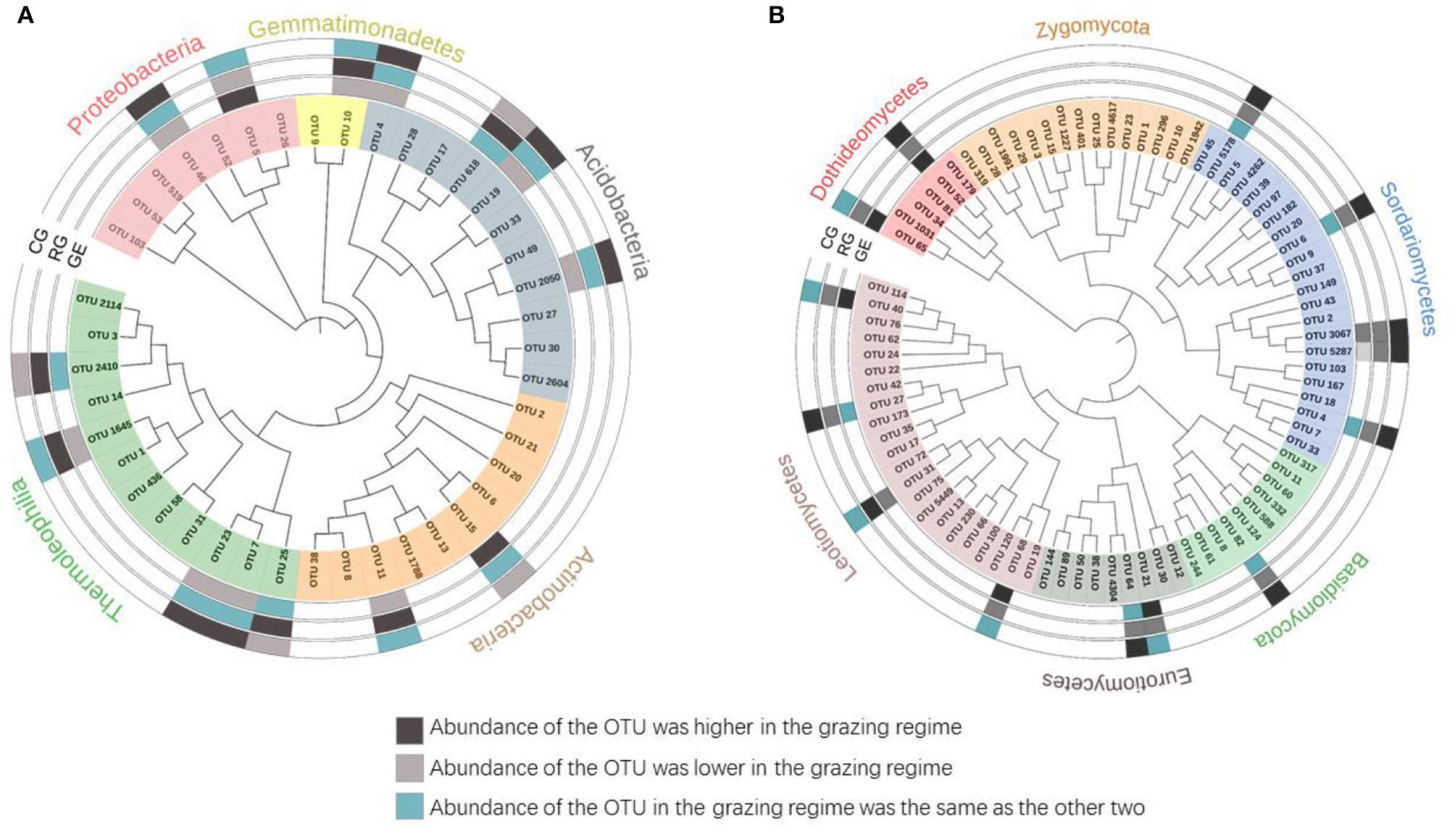
Phylogenetic tree of dominant bacterial **(A)** and fungal **(B)** taxa. GE, grazing exclusion; RG, rotational grazing; and CG, continuous grazing.

To better characterize the grazing regime effect, we identified the dominant taxa (OTUs with a relative abundance ≥ 0.5%). Although 4684 bacterial OTUs and 5241 fungal OTUs were retrieved from all the grazed plots, only 42 (0.8%) and 84 (1.6%) OTUs were identified as the dominant taxa, respectively. For all the grazed plots, these dominant bacterial OTUs were mainly affiliated with Thermoleophilia (28.6%), Actinobacteria (23.8%), and Acidobacteria (26.2%) (Figure 5A); the dominant fungal OTUs were mainly affiliated with Sordariomycetes (26.2%) and Leotiomycetes (26.2%) (Figure 5B). The majority of the dominant taxa did not change with the grazing regime. Specifically, a greater relative abundance of nine dominant fungal taxa was observed under the CG than that under the RG, including OTU 52, OTU 5178, OTU 6, OTU 3067, OTU 5287, OTU 4, OTU 82, OTU 64, and OTU 27, which all belong to Ascomycota. However, a greater relative abundance of six dominant bacterial taxa was observed under the CG than that under the GE, including OTU 46, OTU 4, OTU 19, OTU 2050, OTU 7, and OTU 23.

At the phylum level, soil bacteria were overwhelmingly dominated by Actinobacteria, Proteobacteria, and Acidobacteria; soil fungi were overwhelmingly dominated by Ascomycota, Zygomycota, and Basidiomycota (Supplementary Figure S3). The abundance of Acidobacteria and Armatimonadetes was higher under CG than that under GE, and the abundance of Elusimicrobia was higher under RG and CG than that under GE (Supplementary Figure S4).

### Linkages Among Plant Species and Soil Archaeal, Bacterial, and Fungal Classes

**Figure 6:**
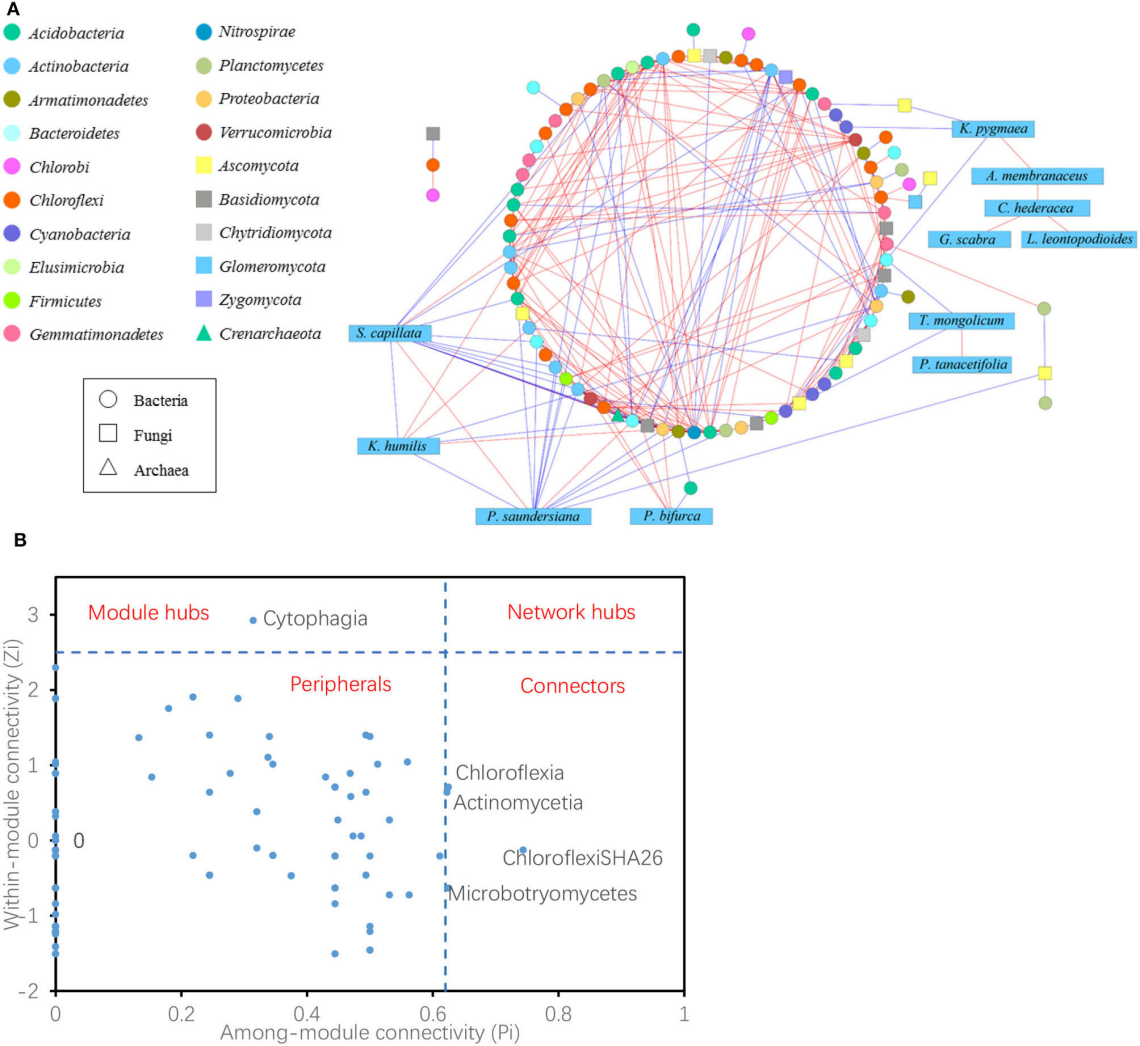
Network interactions of plant species and archaeal, bacterial, and fungal classes **(A)**. A blue edge indicates a negative link between two individual nodes, while a red edge indicates a positive link. Z-P plot showing the distribution of the nodes based on their topological roles. The topological role of each node was determined according to the scatter plot of within-module connectivity (Zi) and among-module connectivity (Pi). The module hubs and connectors were labeled with microbial class names **(B)**.

The co-occurrence network showed 104 nodes, including 76 bacteria, 16 fungi, one archaea, and 11 plant species (Figure 6A). Node with a maximum degree and eigenvector centrality was Deltaproteobacteria, and node with maximum betweenness and stress centrality was Cytophagia. There were 288 links in the network, with 25 negative and 10 positive links between plants and bacteria; four negative links occurred between plants and fungi; one negative link occurred between plants (*S. capillata*) and archaea; 22 positive and 18 negative links occurred between bacteria and fungi; five positive links and one negative link occurred between bacteria and archaea; and one positive link occurred between fungi and archaea. In plant species, *K. pygmaea* is rarely linked with microbes, but it linked four plant specialists that were not directly linked to microbes. *S. capillata* had most links with microbes, 11 with bacteria, one with fungus, and one with archaea, while only two links of them were positive.

Within each group, six positive and two negative links were detected among 11 plant species, whereas 139 positive and 49 negative links were detected among 76 bacterial classes and two positive and two negative links were observed among 16 fungal classes. Overall, for bacteria, Chloroflexi had the most links of 79 among them 62 were positive; for fungi, Ascomycota had the most links of 23 among them 11 were positive.

The topological roles of the nodes identified in the network were shown as a Z-P plot in Figure 6B. The majority (95.2%) of the nodes were peripherals with most of their links inside their modules. Among these peripherals, 46.5% had no links outside their modules (i.e., Pi = 0). Approximately 4.8% of the nodes were generalists, including one module hub and four connectors. However, no network hubs (supergeneralists) were observed in the network. The module hub was Cytophagia, and the connectors were ChloroflexiSHA26, Actinomycetia, Chloroflexia, and Microbotryomycetes. These four of the five microbial generalists showed no difference in their abundances among the grazing regimes (Supplementary Figure S5).

In addition, the Mantel test showed that node connectivity was positively related to the correlation coefficient between plant aboveground biomass, belowground biomass, community coverage, and soil NO_3_-N, while other factors were irrelevant (Supplementary Table S2). Thus, nodes with high connectivity tended to be more sensitive to these factors.

## Discussion

### Effect of Grazing Regime on the Plant and Soil Microbial Composition

Our results found that the aboveground plant community and belowground soil microbial community in the grassland ecosystem showed different responses to grazing disturbance, and the fungi and bacteria in the soil microbial community also behave differently. The grazing regime significantly affected plant community composition and is mainly reflected in two species, *Kobresia pygmaea* and *Stipa capillata*, which are both palatable species for yaks. The former is the dominant species, both GE and CG decreased their coverage compared to RG. However, RG and CG significantly decreased the coverage of *Stipa capillata* compared to GE; *Stipa capillata* therefore can be regarded as a species sensitive to grazing. In contrast, the grazing regime did not alter the soil microbial taxonomic composition, implying a stable soil microbial community in the grazed grassland. For soil microbial diversity, bacterial diversity indices were not affected by grazing regime, while only the Simpson index of the fungal diversity indices had no difference among grazing regimes; these results indicated that the diversity of the bacterial community was more tolerant, while the diversity of fungi was more sensitive to grazing. Previous studies obtained similar results showing that fungal community composition was more sensitive than bacterial community composition to disturbance or management regimes (Lauber et al., 2008) and vegetation change (Dassen et al., 2017) and that there is accumulating evidence that fungi are the first consumers of belowground inputs of plant-derived C (De Deyn et al., 2011; Ballhausen and de Boer, 2016).

The microbial community assembly process is important in coupling community composition with the ecosystem function, which includes two processes: determinism and stochasticity (Zhang et al., 2019). Deterministic processes are based on the theory of ecological niche, stressing the role of environmental filtering imposed by environmental factors in the assembly of the microbial community; stochastic process occurs through ecological drift, including random birth and death and dispersal events (Langenheder and Szekely, 2011). Both deterministic and stochastic processes occur simultaneously during the community assembly and the relative importance changes along with environmental conditions (Tang et al., 2021). We found stochastic mechanisms strongly structured soil microbial community assembly under all grazing regimes. Compared to grazing exclusion, rotational grazing and continuous grazing reduced the relative importance of stochastic ratio, indicating that grazing acted as an environmental filter that increased the deterministic soil microbial community assembly.

The soil bacterial functional composition under RG and CG was similar, while the soil fungal functional composition under GE and RG was similar. From GE to RG, then to CG, the interference increased gradually, implying that the type of interference determines the functional composition of bacteria, while the intensity of interference determines the functional composition of fungi. Aerobic chemoheterotrophy and chemoheterotrophy were the main bacterial functional guilds with rich OTUs, and their relative abundance was higher under GE than that under RG and CG, probably resulting from more organic matter under GE without grazing yak's intakes.

### Effect of Grazing Regime on the Abundance of Soil Microbial Taxa

Only a few soil microbial phyla showed differences in abundance among the grazing regimes. The abundances of Acidobacteria and Armatimonadetes were higher under CG than that under GE, implying a more acidic environment under CG (Bardhan et al., 2012). Acidobacteria is mainly considered oligotrophs and are known to utilize a variety of carbon resources, even recalcitrant carbon substrates, and contribute to litter decomposition and carbon resource conversion, benefiting plant growth (Macdonald et al., 2015; Lladó et al., 2017). Armatimonadetes is related to carbohydrate transportation and metabolism and can also utilize a wide range of carbohydrates (Lee et al., 2014). Continuous removal of plant tissues by yaks under CG reduced plant carbon entering the soil (Supplementary Figure S7), probably stimulating soil microbial carbohydrate transportation and metabolism processes, and leading to the greater abundance of Acidobacteria and Armatimonadetes under CG. Different from our results, Yang et al. (2019) observed an equal relative abundance of Acidobacteria between grazing exclusion, winter grazing, and annual grazing on the Qinghai-Tibet Plateau. Macdonald et al. (2015) found rabbit grazing had a negative impact on the Acidobacteria and thought the result related to rabbit grazing increased C-allocation belowground through root exudation. Two other studies also observed a reduced relative abundance of Acidobacteria under grazing (Xun et al., 2018; Zhang and Fu, 2021). Different results may be related to climate, herbivore type, grazing method, and intensity, thus it is difficult to draw a generalized conclusion about the grazing effects on Acidobacteria. Armatimonadetes received little attention in the studies of grassland grazing, probably because it is not a dominant taxon. At the OTU level, nine dominant fungal OTUs were higher under CG than that under RG, as CG likely benefited slow-growing plant species that produce low-quality litter (high C/N), thus favoring soil fungi; six dominant bacterial OTUs were higher under CG than that under GE, as livestock fed on aboveground plants and returned nearly half of it as feces, thus favoring bacterial growth (Xun et al., 2018). In addition, the grazing increased Acidobacteria and Armatimonadetes that are related to C metabolism, while grazing did not change other microbial phyla.

### Relationships Among Plant Species, Soil Bacteria, Fungi, and Archaea

For bacteria, Chloroflexi is diverse in terms of morphology, nutrition, and metabolic pathways, mediates the biogeochemical cycle of elements such as C, N, and S (Zarzycki et al., 2009; Sorokin et al., 2012, 2014), and had the most links in the network of plant species and soil microbial classes, including 62 positive and 17 negative links. For fungi, Ascomycota, which has the highest species diversity and can break down plant residues, had the most links in the network of plant species and soil microbial classes, including 11 positive and 12 negative links. The module hub of the network was Cytophagia (phylum Bacteroidia), which is an obligate anaerobic gut microbe in animals (Eckburg et al., 2005; Winter and Bäumler, 2014), and it was the hub likely due to the accumulation of dung and urine by grazing (Barik and Murugan, 2015); the connectors were ChloroflexiSHA26, Actinomycetia, Chloroflexia, and Microbotryomycetes. The abundance of four of the five microbial generalists was not affected by the grazing regime, and the little changes may have contributed to the stability of soil microbial composition.

Across plants, bacteria, fungi, and archaea, most of the links between plants and microbes were negative, and they may compete for nutrients, such as available nitrogen, in the infertile environment (Inselsbacher et al., 2010). Plant species were more linked to bacteria than fungi. There were 22 positive and 18 negative links between bacteria and fungi, five positive and one negative links occurred between bacteria and archaea, and only one positive link was detected between fungi and archaea, indicating that bacteria, fungi, and archaea exhibited cooperative interspecies relationships more than competitive interactions. Within each network component, there were six positive and two negative links in the plant community; 73.9% of the bacterial links were positive. Two positive and two negative links were found in 16 fungi, indicating that fungi were more linked with other components than themselves and that intra-group members were more independent. Overall, soil microbes exhibited cooperative behaviors such as cross-feeding, syntrophic interactions, mutualistic interactions, and competition with plant species, similar to intraspecific cooperation and interspecific competition. Microbial communities often act in consortia to synergistically degrade complex plant-derived compounds, with some microbes utilizing metabolites or taking advantage of broken-down products of extracellular enzymes produced by other taxa (Lynd et al., 2002; Alessi et al., 2017).

In the links between plants and microbes, two remarkable plant species were *K. pygmaea* and *S. capillata*. There were few links between *K. pygmaea* and microbes in the network, but it served as a bridge between microbes and four plant specialists that were not directly related to microbes. *S. capillata* was linked with 11 bacteria, one fungus, and one archaea, and most of the links were negative. Coincidentally, *K. pygmaea* and *S. capillata* were also the two special species that had different coverage among grazing regimes. *K. pygmaea* was a grazing-tolerant species and the dominant species in the grazed grassland, while *S. capillata* was a sensitive species that was rarely distributed in the grazed grassland but sharply increased after grazing was prohibited. *K. pygmaea* and *S. capillata* were the two extremes in the plant community, the dominant species *K. pygmaea* acted as a bridge between the plant community and microbes, and the sensitive species *S. capillata* was more independent. It was found that different species play distinct roles in the community and were generally divided into three types. One type was only widely linked to microbes; the other type was plant specialists, which were not directly linked with microbes; and the third type was the bridge between microbes and plant specialists.

Among the environmental factors, plant community aboveground biomass, belowground biomass, coverage, and soil NO_3_-N were closely related to the node connectivity of the network. Nodes with more links tended to be more sensitive to these factors but not to the other factors, such as plant community diversity, soil NH_4_-N, available P, and bulk density. This result implied a close relationship between the soil microbial community and plant community production, in line with previous findings (Van Der Heijden et al., 2008; Yang et al., 2017; Xun et al., 2018; Chen et al., 2020). NO_3_-N is much more mobile than NH_4_-N in soil and can be easily lost by leaching into groundwater and surface runoff (Scherer-Lorenzen et al., 2003); thus, NO_3_-N tends to be the object of competition between plants and microbes and maybe the major cause why many of the links between plants and microbes were negative.

## Conclusion

The grazing regime altered the composition of the plant community but did not change the soil microbial community composition. The network between plant and soil microbial community showed that many plant species were not directly connected with microbes but indirectly connected through dominant species *K. pygmaea*, thus weakening the effect of grazing-induced plant community composition variation on the soil microbial community. Four microbial generalists were not affected by the grazing, hence maintaining the stability of microbial community composition. The stable soil microbial composition is probably also due to the stochastic process dominated by its community assembly. Although soil microbial taxonomic composition was not affected by gazing, its functional composition was significantly changed and GE increased aerobic chemoheterotrophy and chemoheterotrophy in bacteria.

## Data Availability Statement

The original contributions presented in the study are publicly available. This data can be found at: https://www.ncbi.nlm.nih.gov/search/all/?term=PRJNA811340.

## Author Contributions

SD, QG, and CF planned and designed the research. YL, HG, YY, and XG performed experiments, conducted fieldwork, and analyzed data etc. YL and MF wrote the manuscript. All authors contributed to the article and approved the submitted version.

## Funding

This work was financially supported by grants from the National Natural Science Foundation of China (32101315), the China National Key R&D Program (2016YFC0501906 and 2016YFC0502003), the Qinghai Provincial Key R&D Program (2019-SF-145 & 2018-NK-A2), the CTBU high-level talent research start-up project (950319097), and the project of Scientific and Technological Research Program of Chongqing Municipal Education Commission (KJQN20200837).

## Conflict of Interest

YY was employed by China New Era Group Corporation. The remaining authors declare that the research was conducted in the absence of any commercial or financial relationships that could be construed as a potential conflict of interest.

## Publisher's Note

All claims expressed in this article are solely those of the authors and do not necessarily represent those of their affiliated organizations, or those of the publisher, the editors and the reviewers. Any product that may be evaluated in this article, or claim that may be made by its manufacturer, is not guaranteed or endorsed by the publisher.
