# Supplementary material for: Grazing Changed Plant Community Composition and Reduced Stochasticity of Soil Microbial Community Assembly of Alpine Grasslands on the Qinghai-Tibetan Plateau
Source: Front Plant Sci. 2022 May 23;13:864085. doi: 10.3389/fpls.2022.864085 (PMC9168915; doi:10.3389/fpls.2022.864085)
Supplement: Supplementary file 1 [file Data_Sheet_1.pdf]

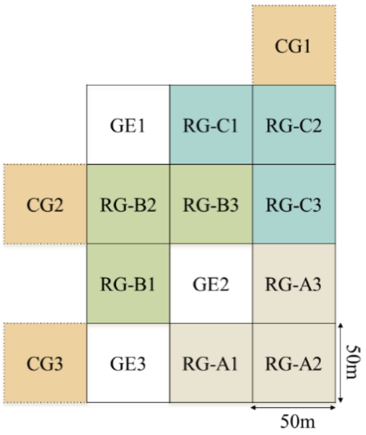

Fig. S1. Plot layout of the grazing regimes. GE, grazing exclusion; RG, rotational grazing; and CG, continuous grazing. RG-A, B, and C were three groups of rotational grazing. 1, 2, and 3 were three replicates of each treatment.

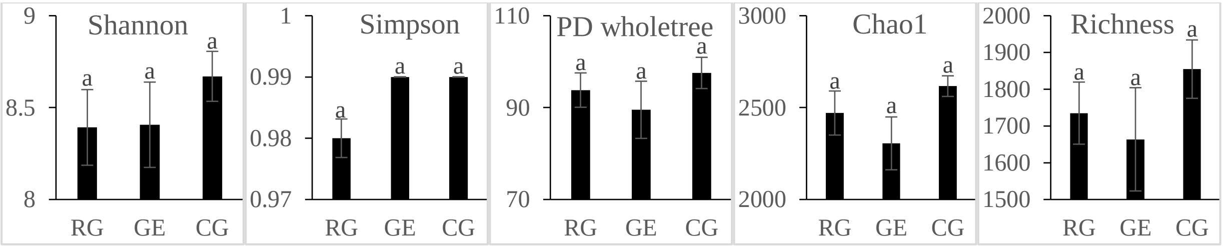

Fig. S2. Soil bacterial diversity among the grazing regimes. GE, grazing exclusion; RG, rotational grazing; and CG, continuous grazing.

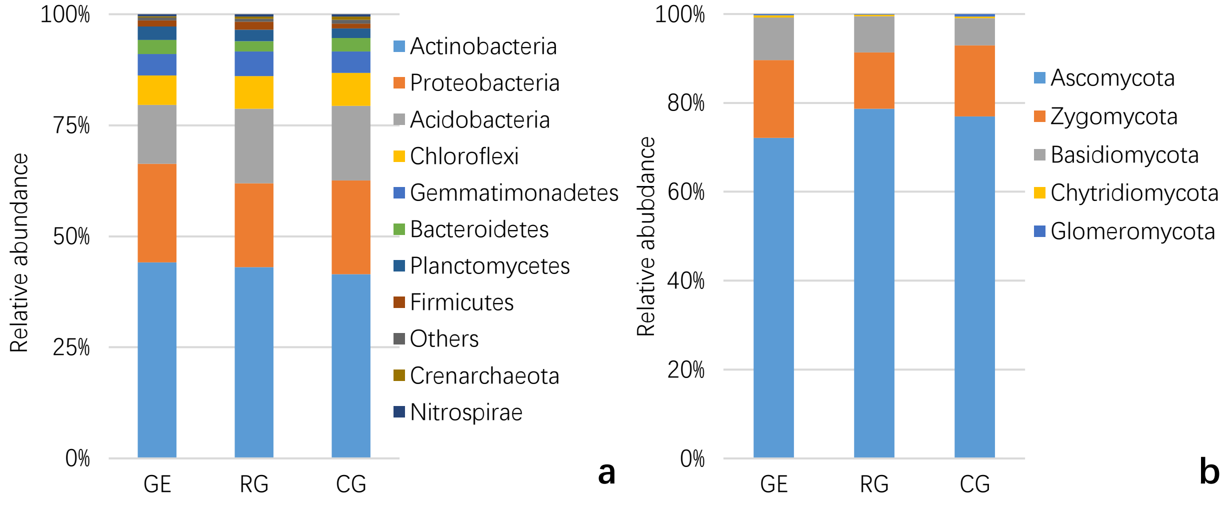

Fig. S3. Relative abundance of phyla for bacterial (a) and fungal (b) communities among grazing regimes. GE, grazing exclusion; RG, rotational grazing; and CG, continuous grazing.

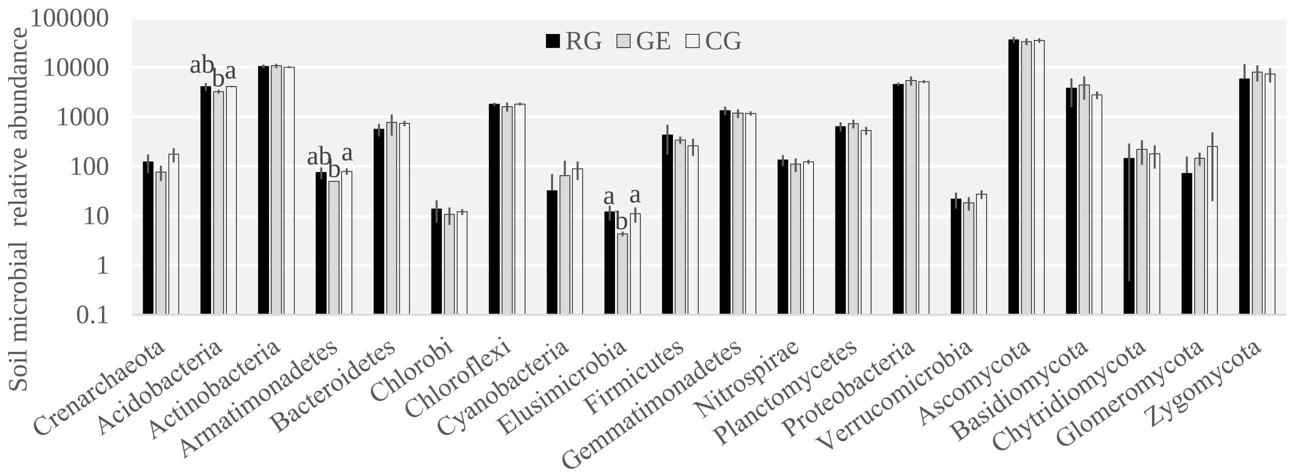

Fig. S4. Relative abundance of soil bacterial, fungal, and archaeal phyla among the grazing regimes. Different lowercase letters above the bars indicate significant differences among the grazing regimes ($p < 0.05$). GE, grazing exclusion; RG, rotational grazing; and CG, continuous grazing.

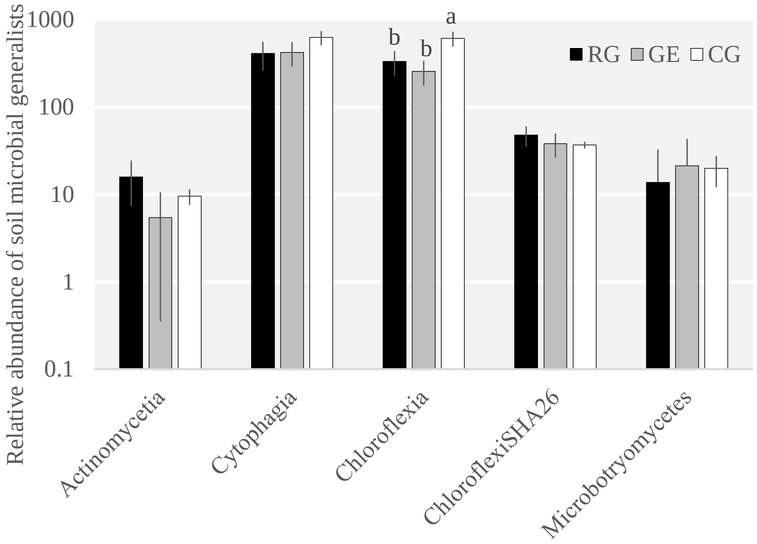

Fig. S5. Relative bundance of five soil microbial generalists among the grazing regimes. Different lowercase letters above the bars indicate significant differences among the grazing regimes ($p < 0.05$). GE, grazing exclusion; RG, rotational grazing; and CG, continuous grazing.

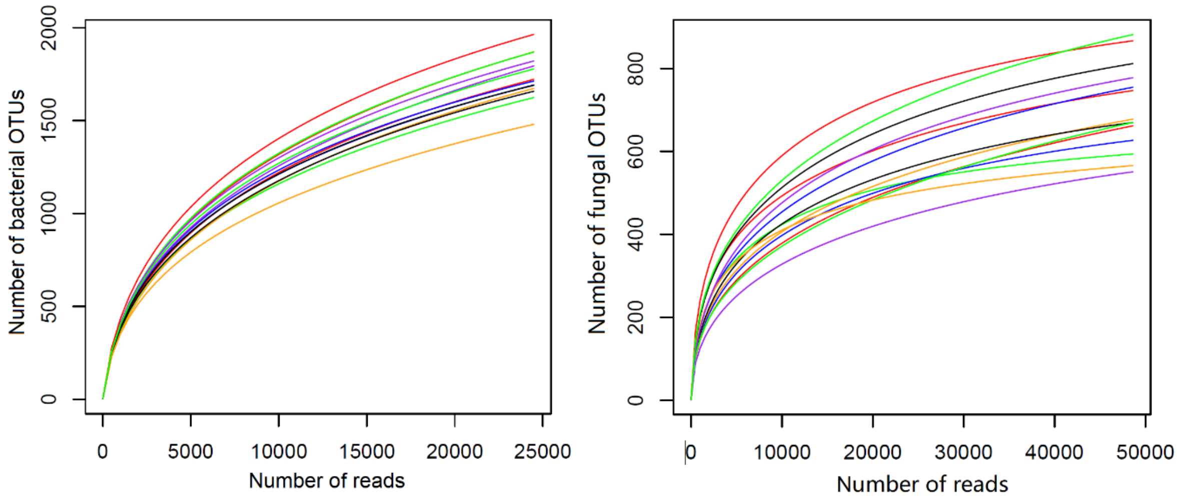

Fig. S6. Rarefaction curves of bacteria and fungi of samples.

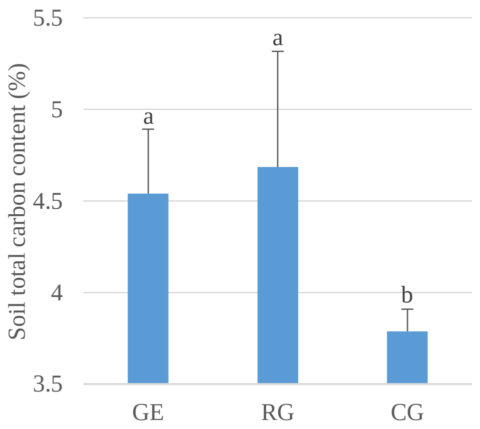

Fig. S7. Soil total carbon content of grazing regimes. Different lowercase letters above the bars indicate significant differences among the grazing regimes ($p < 0.05$). GE, grazing exclusion; RG, rotational grazing; and CG, continuous grazing.

Table S1. Analysis of similarity (ANOSIM) of the plant communities among the grazing regimes

	R	*p*
RG-GE	0.85	0.008
RG-CG	0.45	0.03
GE-CG	0.59	0.1

GE, grazing exclusion; RG, rotational grazing; and CG, continuous grazing.

Table S2. The relationships between the environmental factors and the node connectivity of network of plants and soil microbes

	r	sig
Plant Shannon index	-0.07	0.95
Plant Simpson index	0.002	0.446
Plant richness	0.056	0.143
Plant evenness	0.02	0.318
Plant aboveground biomass	0.325	**0.001**
Plant belowground biomass	0.096	**0.042**
Plant community coverage	0.121	**0.03**
Soil NH_4-N	-0.113	0.999
Soil NO_3-N	0.165	**0.012**
Soil available P	0.003	0.384
Soil bulk density	-0.05	0.858

Table S3. The number of OTU reads of samples.

Sample Name	Bacterial OTU reads	Fungal OTU reads
RGA1	54562	148283
RGA2	63518	58093
RGA3	60035	62523
RGB1	86684	137862
RGB2	27963	141481
RGB3	47627	87587
RGC1	51660	71269
RGC2	56561	142611
RGC3	50299	121950
GE1	55533	49350
GE2	50030	94826
GE3	50521	72256
CG1	46585	48621
CG2	58305	193411
CG3	33627	105826

GE, grazing exclusion; CG, continuous grazing. RGA, RGB, and RGC were three groups of rotational grazing. 1, 2, and 3 were three replicates of each treatment.
